# Benefits of using a support bra in women undergoing coronary artery bypass graft surgery: A randomized trial

**DOI:** 10.1016/j.clinsp.2024.100370

**Published:** 2024-05-20

**Authors:** Thais Rodrigues de Almeida Silva, Julia Nishida Ono, Fabiana Cristina Bazana Remedio Miname, Luís Henrique Wolff Gowdak, Bruno Maher Mioto, Renan Barbosa dos Santos, Luiz Roberto Palma Dallan, Luiz Antonio Machado Cesar

**Affiliations:** Instituto do Coração (InCor), Faculdade de Medicina da Universidade de São Paulo (FMUSP), São Paulo, SP, Brazil

**Keywords:** Quality of life, Surgical support, Pain, Sternotomy

## Abstract

**Background:**

Approximately 30 % of cardiac surgeries are conducted on women by median sternotomy, which often causes discomfort such as pain, affects quality of life, and delayed recovery compared with men. Breast size is related to operative wound complications, such as incisional pain, sternum dehiscence, and infection, which may affect hospital costs due to prolonged hospital stays.

**Objective:**

To evaluate breast size and operative wound complications and the effect of breast support on the incidence of pain, infection, and quality of life in women after coronary artery bypass grafting.

**Method:**

Women were randomly assigned to one of three groups: group A (surgical breast support), group B (ordinary breast support), and group C (no-support). Observations were taken daily between the second and seventh postoperative days and at 30, 60, and 180 days. Pain was assessed using the Short-Form 36 Health Survey (SF36) for quality of life and a verbal numerical scale. The authors used the nonparametric Kruskal–Wallis and Friedman tests to examine variance. The authors used the Pearson correlation coefficient or the Spearman correlation for correlations between variables. A multivariate study was conducted to evaluate the occurrence of infection, and the logistic regression model with “stepwise” variable selection was used. A linear regression model with the “stepwise” variable selection was also used for hospitalization. The authors used SPSS 17.0 software for Windows, with a significance level of *p* < 0.05.

**Results:**

There was no difference in pain evaluation between the groups in 190 women (*p* > 0.05). When comparing quality of life, there was a statistically significant difference in the functional capacity domain at 30 and 60 days, with group A having the best functional capacity (*p* < 0.05). The larger the breast size, the longer the hospital stay (*p* < 0.001) and the higher the probability of infection (*p* = 0.032). Patients with a history of stroke had a 3.8 higher incidence of infection (*p* = 0.040).

**Conclusion:**

The use of surgical support did not affect acute pain or sternal infection rate in the 6-month follow-up. However, it was effective in the functional capacity domain 30 days after surgery and maintained at 60 days.

## Highlights

• The larger the breast size, the longer the hospital stay and the higher the probability of infection.
• Patients with a history of stroke had a higher incidence of infection.
• The use of surgical support can improve quality of life.

**What is already known**

Women undergoing coronary artery bypass graft surgery have a higher mortality rate and greater complications than men during the in-hospital phase.

Larger breasts are associated with a higher rate of thorax surgical wound infection and longer hospital stays.

The use of surgical support improves recovery from cardiac surgery, reducing pain and decreasing sternum instability, with reduced sternal wound infection.

Alt-text: Unlabelled box

**What this paper adds**

Surgical support did not prevent wound infections in the patients, but it improved their quality of life, allowing them to return earlier to their daily activities.

Women with larger breasts had a higher quality of life in terms of chest pain at 60 days and functional capacity and vitality at 180 days.

Alt-text: Unlabelled box

## Introduction

Individuals with coronary artery disease may benefit from a Coronary Bypass Graft (CABG),^1^ and sternotomy is used to access the pathway.^2^ Performing this type of surgery aims to improve life expectancy and quality of life and relieve pain caused by ischemia.^3^ Although there are some differences in cardiovascular risk factors between women and men, the same is true for results after revascularization.^3^, ^4^, ^5^, ^6^, ^7^, ^8^, ^9^, ^10^, ^11^ Women have more pain and discomfort related to sternotomy than men.^12^

Since the 1990s, many studies have suggested that it would be better to use a specific bra support to avoid infection.^9^^,^^11^, ^12^, ^13^, ^14^ The most recent trial was published in 2013 by Gorlitzer et al.,^15^ a randomized multicenter study that concluded favorably the use of a bra to avoid these complications, as well as the possible benefits of support for men, implying that it would be helpful for those with a body mass index greater than 35 kg/m^2^.^16^

Therefore, this study aimed to evaluate the effect of bra support immediately after CABG in the public health system hospital population as a preventive measure to minimize mediastinitis, superficial sternal infections, and pain and improve quality of life months after surgery. If this is the case, it will most likely be cost-effective and included as part of the standard care for these patients.

## Methods

The authors conducted a randomized, nonblinded trial on women undergoing CABG at the Heart Institute of the Clinical Hospital of the University of São Paulo.

### Primary objective

The primary objective of this study was to compare the incidence of chest pain in women undergoing coronary artery bypass grafting with and without breast support.

### Secondary objectives

The secondary objectives of this study are to compare quality of life, incidence of infection/operative wound dehiscence, and use of analgesics and antibiotics between groups during hospitalization and 30, 60, and 180 days after surgery.

The randomization was achieved after participants signed the consent form using a random list provided by a particular randomization website.^17^

### Inclusion criteria

This study included women over the age of 18 yr. of age undergoing CABG with a median sternotomy.

### Exclusion criteria

Women with previous breast surgery (mammoplasty or mastectomy), thoracic radiotherapy, any altered cognitive level, or who have been in the postoperative intensive care unit for more than 7 days were excluded from the study.

### Intervention model

There is no protocol in place for the use of particular breast support after cardiac surgery. The authors occlude the surgical wound with curative agents for 48 h and then leave it open if no secretion is present. If secretion appears, the authors clean the wound with an occlusive sterile dressing and close the incision with sterile gauze and transparent film. As a result, a pilot experiment was performed with 20 patients, to whom we supplied four different models of surgical support bras. Each patient selected the one that provided the most comfort and safety.

### Study design

The surgical support bra (group A), regular support (group B), and no-support (group C) were randomly assigned in a 1:1:1 ratio.

Patients were required to sign a consent form and complete two questionnaires at the time of selection: identification and characterization of the patient and quality-of-life SF36, which was validated for the Portuguese language in 1999.^18^ Patients were instructed to use the bra for 24 h a day or for as long as they could handle daily until the end of the study. Those randomized to the no-support group were also instructed to avoid wearing bras. The wound was evaluated on the first day of admission to the ward and continued daily until hospital discharge. The degree of pain and the use of analgesics and antibiotics were recorded. Pain was assessed in two methods: (a) Using the SF36 before surgery and at 30, 60, and 180 days after surgery and (b) Especially for the thoracic wound, daily, during hospitalization, and at 30, 60, and 180 days after surgery. A numerical verbal scale ranging from 0 to 10 was used for this objective. Patients were instructed to gradually return to their normal activities upon hospital discharge, according to a postoperative guidebook provided.

Patients were withdrawn from the study if they experienced any of the following: (a) Use of negative pressure therapy in the thorax or leg to treat infection, (b) Length of postoperative stay in the intensive care unit longer than 7 days, (c) Readmission to the intensive care unit with a stay longer than 3 days, (d) Length of stay in the ward longer than 20 days after surgery, and (e) Readmission to the hospital with a hospital stay of ≥10 days.

This study was approved by the Research Ethics Committee of Clinical Hospital, School of Medicine, University of São Paulo (n° 38,513,214.8.0000.0068), on September 19, 2014, it is registered at clinicaltrials.gov (NCT02864186), and it is a accordance with the CONSORT Statement rules.

### Sample size calculation

To calculate the sample size, the authors used a study that found that 86.7 % of patients experience pain after cardiac surgery.^19^ Assuming a 25 % reduction in pain using a surgical support bra, the authors would require 42 patients in each group to achieve a difference with 5 % significance and 80 % power to detect differences between strategies. The authors would need 126 patients, including all follow-ups.^20^ Assuming a 20 % loss due to intraoperative and immediate postoperative deaths, withdrawal during follow-up, and changes in therapeutic conduct, the sample size was 152 patients.

### Statistical analysis

All variables were initially analyzed. Quantitative variables were analyzed by monitoring minimum and maximum values and means, standard deviations, and quartiles. For qualitative variables, absolute and relative frequencies were calculated.

The authors analyzed variance with repeated measures to compare visits in each group.^21^ When the normality assumption was rejected, we used the nonparametric Kruskal-Wallis test^22^ and Friedman test.^23^ The normality of data was determined using the Kolmogorov-Smirnov test.^23^ The authors used the analysis of variance to a factor with the Bonferroni test to compare the three groups.^23^ When the assumption of data normality was rejected, the authors used the nonparametric Kruskal-Wallis^22^ test in conjunction with the Dunn test. The Chi-Square test^22^ or the Fisher exact test^22^ was used to evaluate proportional homogeneity. The authors used the Pearson correlation coefficient^22^ or the Spearman correlation coefficient^22^ for correlations between variables (when the assumption of normality was rejected). The authors used the logistic regression model with the “stepwise” variable selection process for the multivariate study of the variables associated with the occurrence of infection.^23^ The authors used the linear regression model with the “stepwise” variable selection process^24^ for the hospitalization. The SPSS 17.0 software for Windows was also used.

## Results

**Fig. 1:**
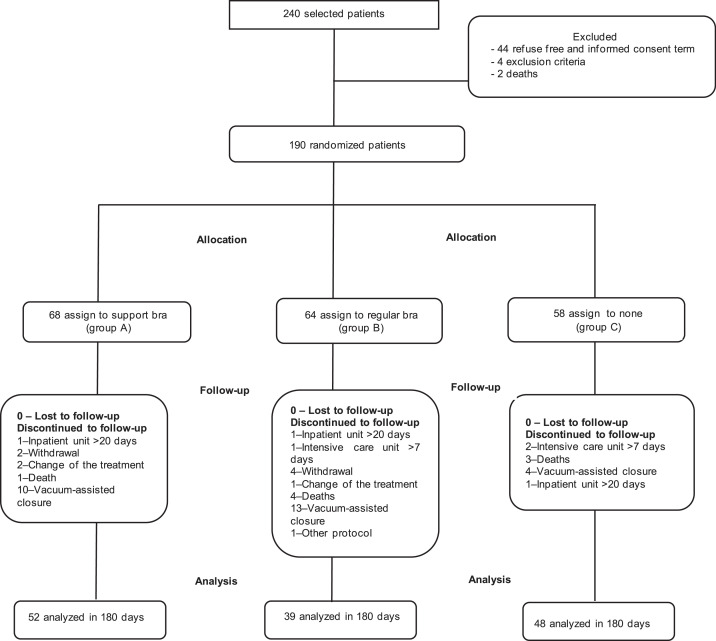
The consolidated standards of reporting trial diagram.

From January 2015 to September 2018, 240 women undergoing CABG were examined. Four patients were excluded because they did not meet the inclusion criteria, and 44 patients refused to participate; thus, 192 patients gave consent and were included in the study. Two patients died before randomization; therefore, 190 patients were randomized (Fig. 1).

### Characteristics

The majority of the 190 women were White (75.26 %), Catholic (62.11 %), and had completed elementary school (66.76 %). The mean age was 63 yr., and 38.42 % were married. This sample had a homogeneous distribution in relation to comorbidities, weight, height, graft types, extracorporeal circulation time, and anoxia. The majority had diabetes (55.5 %), used metformin (40 %), had systemic arterial hypertension (89.7 %), and dyslipidemia (63.2 %) (Table 1). In terms of CABG indications, 77 (40.7 %) were elective, and 112 (59.3 %) were urgent. The median hospitalization period for the three groups was 5.0 days, counting from admission to the surgical procedure. The median hospital stay time for group A was 12.5 days, 14 days for group B, and 15 days for group C. Most surgeries were performed with extra body circulation with an average time of 88.91 ± 25.82 min, and revascularization was performed with an arterial graft (82.1 %) with or without one or two venous grafts (48.4 %).

**Table 1:** Baseline characteristics of the population in each group.

Variable	*n* = 90	Group A (*n* = 68)	Group B (*n* = 64)	Group C (*n* = 58)	P
Clinical and perioperative characteristics					
Age	63 ± 8	62.79 ± 7.81	62.89 ± 8.50	63.48 ± 9.44	0.891^a^
Ethnic group (White)	143 (75.3 %)	54 (79.4 %)	49 (76.6 %)	40 (69.0 %)	0.743^b^
Anthropometric measurements					
Weight	68.81±12.77	69.25±11.80	70.80±13.19	66.08±13.15	0.117^a^
Height	155.31±5.92	155.04±5.57	156.02±6.00	154.83±6.26	0.492^a^
BMI	28.54±5.13	28.92±5.46	29.02±4.61	27.57±5.22	0.219^a^
Circumf. infra	92.59±8.10	92.52±7.83	93.30±8.56	91.89±7.98	0.631^a^
Bust	106.24±10.93	106.38±9.75	108.20±12.44	103.92±10.18	0.096^a^
Comorbidity					
DM	106 (55.8 %)	37 (54.4 %)	40 (62.5 %)	29 (50.0 %)	0.366^a^
SAH	164 (86.3 %)	61 (89.7 %)	53 (82.8 %)	50 (86.2 %)	0.515^a^
DLP	120 (63.2 %)	45 (66.2 %)	38 (59.4 %)	37 (63.8 %)	0.715^a^
Smoker	37 (19.5 %)	8 (11.8 %)	18 (28.1 %)	11 (19.0 %)	0.060^a^
Ex-smoker	61 (32.1 %)	23 (33.8 %)	17 (26.6 %)	21 (36.2 %)	0.486^a^
Previous stroke	11 (5.8)	7 (10.3 %)	3 (4.7 %)	1 (1.7 %)	0.135^b^
AMI	84 (44.2 %)	28 (41.2 %)	27 (42.2 %)	29 (50.0 %)	0.563^a^
Antidiabetic medication					
Sulfonylurea	36 (19 %)	13 (19.1 %)	13 (20.3 %)	10 (17.2 %)	0.910^d^
Metformin	76 (40 %)	28 (41.2 %)	28 (43.8 %)	20 (34.5 %)	0.563^d^
Inib_DPP4	8 (4.2 %)	5 (7.4 %)	3 (4.7 %)	0 (0.0 %)	0.122^b^
Pioglitazone	5 (2.6 %)	2 (2.9 %)	2 (3.1 %)	1 (1.7 %)	1.000^b^
Insulin	34 (17 %)	14 (20.6 %)	13 (20.3 %)	7 (12.1 %)	0.381^b^
Without medication	11 (5.8 %)	3 (4.4 %)	5 (7.8 %)	3 (5.2 %)	0.736^b^
Gateway					0.226^b^
Elective	77 (40.7 %)	24 (35.8 %)	32 (49.2 %)	21 (36.8 %)	
Urgent	112 (59.3 %)	43 (64.2 %)	33 (50.8 %)	36 (63.2 %)	
Perioperative characteristics					
CPB	159 (85.03 %)	54 (81.8 %)	56 (88.9 %)	49 (84.5 %)	0.526^d^
Type of graft					
Arterial	177 (93.2 %)	64 (94.1 %)	60 (93.8 %)	53 (91.4 %)	0.822^d^
Venous	162 (85.3 %)	54 (79.4 %)	54 (84.4 %)	54 (93.1 %)	0.094^d^

^c^ Descriptive probability level of Likelihood Ratio Test.

^d^ Descriptive level of probability of the Chi-Square test.

AMI, Acute myocardial Infarction; BMI, Body Mass Index; Circumf infra, Circumference below the breast; CPB, Extracorporeal Circulation; DLP, Dyslipidemia; DM, Diabetes Mellitus; Inib_DPP4, Inhibitors of the enzyme Dipeptidyl Peptidase-4; SAH, Systemic Arterial Hypertension.

a Descriptive level of probability of one-factor analysis of variance.

b Descriptive level of probability of Fisher exact test.

There was no statistically significant difference between groups in terms of absolute and relative pain frequencies during hospitalization at visits, 30 days (*p* = 0.386), 60 days (*p* = 0.207), and 180 days (*p* = 0.547) after surgery, as well as the use of painkillers (Table 2).

**Table 2:** Absolute and relative frequencies of the presence of pain and the use of medication, according to the study group.

	Groups			
	Group A	Group B	Group C	p
**Pain**	27 (41.5 %)	23 (37.7 %)	26 (47.3 %)	0.578^a^
**Painkillers**				
Dipyrone	24 (88.9 %)	21 (91.3 %)	22 (84.6 %)	0.824^b^
Acetaminophen	1 (3.7 %)	0 (0.0 %)	4 (15.4 %)	0.098^b^
Opioid	2 (7.4 %)	4 (17.4 %)	1 (3.9 %)	0.279^b^
Without medication	2 (7.4 %)	2 (8.7 %)	0 (0.0 %)	0.452^b^

a Descriptive level of probability of the Chi-Square test.

b Descriptive probability level of Fisher exact test.

The Friedman test revealed that all three groups (had significant changes during follow-up in the SF36 functional capacity domain (Table 3). Furthermore, all the domains of the SF36 questionnaire evaluated showed a difference between groups during the follow-up (*p* < 0.001), except for *p* = 0.237. The nonparametric Kruskal-Wallis test showed that the groups did not differ significantly in the preoperative and at 30, 60, and 180 days (*p* > 0.05). When the correlation between breast size and SF36 domains of quality of life was examined, a weak negative but significant correlation was found between breast size and functional capacity (*p* = 0.018) score at 60 days and pain (*p* = 0.006) and vitality (*p* = 0.049) scores at 180 days (Supplementary Table 1).

**Table 3:** Descriptive values of the SF36 functional capacity domain based on the time of assessment and groups.

Groups	Moment	Average	SD	Minimum	Maximum	P_25_	Median	P_75_	p	Dunn^a^(p)
	Specific support group									
	Before	36.73	32.96	0	100	6.25	20	65	<0.001	<0.005
	30 days	67.5	20.59	25	100	46.25	70	85		
	60 days	81.35	17.6	25	100	71.25	85	95		
	180 days	77.4	24.94	0	100	61.25	90	95		
	**Regular support group**									
	Before	41.03	31.94	5	100	10	30	65	<0.001	
	30 days	62.44	23.64	10	100	45	65	80		
	60 days	76.03	20.1	15	100	65	85	90		
	180 days	81.54	23.12	10	100	70	90	100		
	**Group without support**									
	Before	31.33	28.97	0	100	10	20	40	<0.001	
	30 days	53.67	21.33	5	95	37.5	55	70		
	60 days	70.61	20.45	20	100	57.5	80	85		
	180 days	77.86	25.58	0	100	67.5	85	95		

a DUNN method.

There was no statistically significant difference when the absolute and relative frequencies of complications associated with the number of thoracic wound infections were compared (*p* = 0.069, Table 4).

**Table 4:** Absolute and relative frequencies of postoperative complications based on the study group.

	Group A (*n* = 68)		Group B (*n* = 64)		Group C (*n* = 58)		
Complications	n	%	n	%	n	%	p
Other infections	12	17.7	13	20.3	9	15.5	0.786^a^
Thorax wound infection	18	26.5	20	31.3	8	13.8	0.069^a^

a Descriptive level of probability of the Chi-Square test.

There were no statistically significant differences in the presence or absence of infection related to the size of the breasts between groups (Supplementary Table 2) and the length of hospital stay and thoracic wound infection, but group C (without breast support) had a longer hospitalization time (Supplementary Table 3).

The authors found that breast size is associated with the length of hospital stay when we performed a multivariate analysis of variables diabetes mellitus, dyslipidemia, chronic kidney failure, smoking, previous stroke, cardiac failure, acute myocardial revascularization, breast size, body mass index, ventricular function, and age using a stepwise selection process (*p* < 0.001). Furthermore, breast size and previous stroke are associated with the occurrence of infection. Therefore, the larger the breast size, the higher the risk of infection (*p* = 0.032). Patients who had previously experienced stroke have a 3.8 times higher incidence (95 % CI 1.07–13.70) of infection than those who had not previously experienced stroke.

## Discussion

The authors were unable to demonstrate that using a breast support bra has a significant effect on incisional pain, infection, and quality of life in women who underwent myocardial revascularization surgery. Our data showed no significant differences in pain, both in the general case when the pain domain related to quality of life was evaluated and in the specific aspect when thoracic pain was present during hospitalization, from the fifth to seventh postoperative day and after hospital discharge (30, 60, and 180 days).

Pain, on the other hand, is a common complaint in the postoperative period of myocardial revascularization surgery, and even with the available medical arsenal for its treatment, it is still considered a concern for women undergoing CABG. It is important to note that without adequate attention to the patient's pain, it might result in intense suffering and risk exposure. Pain is classified as acute and represents a social, economic, and health problem that is relieved in less than 30 % to 50 % of adult and pediatric patients.^25^ Treatment of pain is an important factor in faster recovery and is intrinsically linked to improving a patient's quality of life. Not to mention that pain relief associated with comfort results in significant improvements in physical, mental, and social status.^26^

In this study, pain was explicitly analyzed for the thorax region. However, it was impossible to demonstrate the benefits of a support bra contrary to reports in the literature that link the onset of pain to breast size^16^^,^^27^^,^^28^ and recommend the use of breast support for pain management^29^ and mentioning that the use of support would improve the pain related to the breast but not the incisional pain proper.^30^

The authors had no cases of mediastinitis, most likely due to early diagnosis and treatment of surgical wound infection, probably because we had a plastic surgeon with us during all hospital stays to evaluate any suspected local infection. Although the study suggests that using a strap/vest prevents sternum instability, dehiscence, and mediastinitis,^15^^,^^28^^,^^31^ the authors believe that our wound surveillance prevented mediastinitis by treating an early superficial sternal wound infection. Three studies have demonstrated an incidence of surgical wound complications by comparing the use of a surgical vest with not using one. Two studies included more than 1500 individuals, with an average age of 67 yr., from both sexes, who underwent any type of cardiac surgery, and the incidence of deep and superficial infection was 2 %–3 %.^15^^,^^28^ The third study included 310 individuals. The authors only evaluated mechanical sternal complications with the same patient profile as in other studies and found minor mechanical complications, shorter hospitalization time, less use of analgesics, less pain, and higher quality of life, although the latter visit was only for a single moment.^32^ Despite the fact that our sample had significantly younger patients (62-years-old), it had a higher percentage of diabetes and exclusively women, which makes a difference when compared with the valve and other cardiac surgeries. The authors would predict even more infections because our patients had atherosclerosis and diabetes, which affect wound healing.

Indeed, the authors found a higher number of infections as the size of the breast increases. The multivariate analysis using the linear regression model revealed that the larger the breast, the longer the hospitalization time and the more frequently infection occurred. This supports the literature, which shows that breasts of medium and large sizes impose considerable inferolateral tension in the sternotomy midline,^10^^,^^13^ favoring the occurrence of dehiscence and the possibility of infection,^3^^,^^10^^,^^13^^,^^14^ resulting in an increase in hospitalization time. Another issue is that the authors found no study in the literature that addressed the issue of breast size and quality of life (SF36) at 30, 60, and 180 days, which is a new finding of our trial.

A recent study examined the conformity of use and satisfaction when using three models of bra support, one of which was the one used in our study.^33^ However, the authors did not have 100 % adherence from our participants, which poses a challenge. Therefore, the role nurses play in this context is essential for contributing to the care provided to the hospitalized patient because nurses are the professionals who stay 24 h at the patient's bedside, and the initial information and input to the use will always come from them.

Aside from the fact that women are underrepresented in research, studies including this method as a tool to reduce complications and increase patient satisfaction are limited in the literature. Then, more research with both sexes should be conducted to address this treatment approach for patients more appropriately.^34^

### Limitations

This study has some limitations. First, the authors did not use the breast size as an inclusion criterion to evaluate the pain and infection aspects of thoracic wound surgery. Second, follow-up bias may exist because the intervention depends on the patient's adherence and commitment. Even when the importance of the intervention is explained to the patient, it cannot be guaranteed that dedication is completely performed.

## Conclusion

In our study population, using a surgical support bra did not alleviate postoperative pain; however, it was effective in the functional capacity domain after 30 and 60 days. There was no correlation between the use of surgical support and the sternal infection rate. Second, the size of the breast was associated with the appearance of infection and impacted the length of hospitalization with a lower quality of life. The larger the breasts, the lower the quality of life after coronary artery bypass graft surgery.

## Authors' contributions

Thais Rodrigues de Almeida Silva: Conceptualization, methodology, investigation, formal analysis, visualization, writing – original draft, writing – review & editing.

Julia Nishida Ono: Comceptualization.

Fabiana Bazana Remedio Miname: Writing – review & editing.

Luís Henrique Wolff Gowdak: Formal analysis, writing – review & editing.

Bruno Maher Mioto: Writing – review & editing.

Renan Barbosa dos Santos: Writing – review & editing.

Luiz Roberto Palma Dallan: Writing – review & editing.

Luiz Antonio Machado Cesar: Supervision, conceptualization, methodology, writing – review & editing. final approval of the article.

## Declaration of competing interest

The authors declare that they have no known competing financial interests or personal relationships that could have appeared to influence the work reported in this paper.

## Appendix Supplementary materials

Image, application 1
