# Supplementary material for: Benefits of using a support bra in women undergoing coronary artery bypass graft surgery: A randomized trial
Source: Clinics (Sao Paulo). 2024 May 20;79:100370. doi: 10.1016/j.clinsp.2024.100370 (PMC11134560; doi:10.1016/j.clinsp.2024.100370)
Supplement: Supplementary file 1 [file mmc1.docx]

**CLINICS-D-23-00278_Supplementary Material**

**Supplementary Table 1** Spearman correlation coefficient between breast size and SF36 domains at 30, 60, and 180 days.

| **Domain SF36** |  | **30-days** | **60-days** | **180-days** |
| --- | --- | --- | --- | --- |
| **Functional capacity** | *r* | -0.083 | -0.199 | -0.146 |
|  | *p* | 0.316 | 0.018 | 0.084 |
| **Physical aspects** | *r* | -0.006 | -0.019 | -0.109 |
|  | *p* | 0.945 | 0.825 | 0.197 |
| **Pain** | *r* | -0.154 | -0.158 | -0.232 |
|  | *p* | 0.062 | 0.061 | 0.006 |
| **General state health** | *r* | 0.055 | 0.007 | -0.068 |
|  | *p* | 0.503 | 0.937 | 0.426 |
| **Vitality** | *r* | -0.142 | -0.135 | -0.166 |
|  | *p* | 0.086 | 0.110 | 0.049 |
| **Social aspects** | *r* | -0.113 | 0.026 | -0.044 |
|  | *p* | 0.171 | 0.762 | 0.607 |
| **Emotional aspects** | *r* | -0.045 | 0.010 | -0.128 |
|  | *p* | 0.583 | 0.904 | 0.129 |
| **Mental health** | *r* | -0.047 | -0.129 | -0.141 |
|  | *p* | 0.571 | 0.127 | 0.094 |

**Supplementary Table 2** Mean and standard deviation of the size of the breast based on the study group and thorax wound infection.

| **Group** | **Thorax wound infection** | | **Total** |
| --- | --- | --- | --- |
|  | **Without infection** | **With a thorax wound infection** |  |
| A | 104.94±9.37 | 110.39±9.90 | 106.38±9.75 |
| B | 106.03±12.18 | 113.93±10.70 | 108.50±12.22 |
| C | 103.19±9.23 | 108.50±14.78 | 103.92±10.18 |
| **Total** | 104.67±10.26 | 111.60±11.14 |  |

(*) Descriptive level of probability of the Fisher exact test.

**Supplementary Table 3** Descriptive values of length of stay based on study group.

| **Thorax wound infection** | **Group** | | |  |
| --- | --- | --- | --- | --- |
|  | **A** | **B** | **C** | **p^a^** |
| Yes | 21.41±17.57 | 19.10±13.87 | 36.25±21.31 | 0.150 |
|  | Med = 15.00 | Med = 15.00 | Med = 18.00 |  |
| No | 17.17±7.75 | 20.96±21.59 | 16.63±6.84 | 0.976 |
|  | Med = 16.00 | Med = 15.00 | Med = 15.00 |  |
| p^b^ | 0.895 | 0.921 | 0.064 |  |

^a^ Descriptive probability level of the Kruskal-Wallis nonparametric test.

^b^ Descriptive probability level of the Mann-Whitney *U* test.
